# Mixed lineage kinases activate MEK independently of RAF to mediate resistance to RAF inhibitors

**DOI:** 10.1038/ncomms4901

**Published:** 2014-05-22

**Authors:** Anna A. Marusiak, Zoe C. Edwards, Willy Hugo, Eleanor W. Trotter, Maria R. Girotti, Natalie L. Stephenson, Xiangju Kong, Michael G. Gartside, Shameem Fawdar, Andrew Hudson, Wolfgang Breitwieser, Nicholas K. Hayward, Richard Marais, Roger S. Lo, John Brognard

**Affiliations:** 1 Signalling Networks in Cancer Group, Cancer Research UK Manchester Institute, The University of Manchester, Manchester M20 4BX, UK; 2 Division of Dermatology, Department of Medicine, Jonsson Comprehensive Cancer Center and the University of California, Los Angeles, California 90095-1750, USA; 3 Molecular Oncology Group, Cancer Research UK Manchester Institute, The University of Manchester, Manchester M20 4BX, UK; 4 Oncogenomics Research Group, QIMR Berghofer Medical Research Institute, Herston, Brisbane QLD 4006, Australia; 5 Cell Regulation Group, Cancer Research UK Manchester Institute, The University of Manchester, Manchester M20 4BX, UK; 6 These authors contributed equally to this work

## Abstract

RAF inhibitor therapy yields significant reductions in tumour burden in the majority of V600E-positive melanoma patients; however, resistance occurs within 2–18 months. Here we demonstrate that the mixed lineage kinases (MLK1–4) are MEK kinases that reactivate the MEK/ERK pathway in the presence of RAF inhibitors. Expression of MLK1–4 mediates resistance to RAF inhibitors and promotes survival in V600E-positive melanoma cell lines. Furthermore, we observe upregulation of the MLKs in 9 of 21 melanoma patients with acquired drug resistance. Consistent with this observation, MLKs promote resistance to RAF inhibitors in mouse models and contribute to acquired resistance in a cell line model. Lastly, we observe that a majority of MLK1 mutations identified in patients are gain-of-function mutations. In summary, our data demonstrate a role for MLKs as direct activators of the MEK/ERK pathway with implications for melanomagenesis and resistance to RAF inhibitors.

## Abstract (web summary)

B-Raf is mutated in many melanomas but treatment of the disease with small molecules targeting the mutant protein often results in tumour resistance. Here, the authors show that mixed lineage kinases (MLK1-4) can reactivate the B-Raf signalling pathway in the presence of inhibitors, resulting in drug resistance.

## Graphical abstract

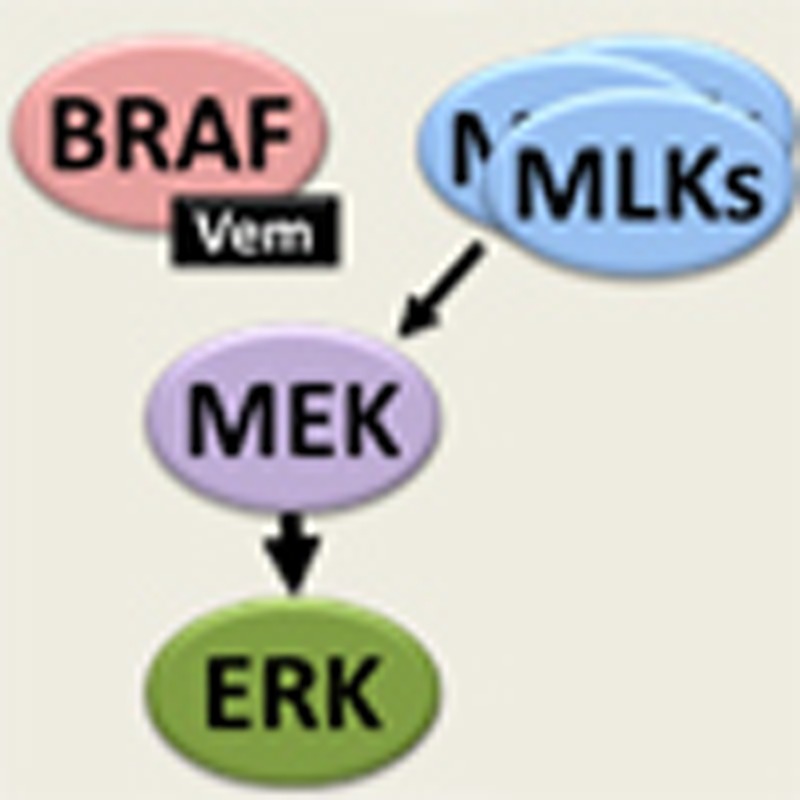

The MLKs are MAP3Ks that regulate both the JNK and p38 MAPK pathways^1^. They directly phosphorylate MKK4/7 to activate the JNK pathway and MKK3/6 to activate the p38 pathway in response to extracellular stimuli, leading to regulation of a diverse array of cellular fates^1^. The MLK family contains primary family members (MLK1–4, also known as *MAP3K9*, *MAP3K10*, *MAP3K11* and *KIAA1804*, respectively) that have Src homology-3 (SH3) and CRIB (Cdc42/Rac-interactive binding region) domains, and subfamily members (DLK, LZK and ZAK) that lack these regulatory domains^1^. Previous studies have indicated that one family member (MLK3) can activate the RAF/MEK/ERK pathway; however, this activation appeared independent of kinase activity and the authors suggested MLK3 acted as a scaffold to promote BRAF activation^2^,^3^. Consistent with MLK3 regulating the RAF/MEK/ERK pathway, depletion of endogenous MLK3 suppressed activation of the pathway, and mice deficient in both MLK2 and MLK3 have reduced activation of ERK in response to tumour necrosis factor stimulation^2^,^4^. In addition, various studies suggest that MLKs are oncogenic and mutations in this family of kinases have been observed in several different cancers^5^,^6^,^7^. *MAP3K9* (MLK1) has been identified as a gene that is frequently mutated in melanoma (12 of 85, or 14%, of melanoma patients evaluated had MLK1 mutations)^8^. Recently, genetic alterations in MLKs have been reported by cancer genomics data sets at a frequency of 15, 18 and 25% in cutaneous skin melanomas^9^,^10^,^11^,^12^. However, the role of the MLKs in melanomagenesis or resistance to RAF inhibitors has not been investigated to date.

Aberrant activation of the MEK/ERK pathway leads to tumorigenesis and the role of mutationally activated BRAF as a driver of metastatic melanoma has been well established^13^,^14^,^15^. Inhibition of mutationally activated BRAF^V600E^ by vemurafenib or dabrafenib results in significant clinical response rates in V600E-positive metastatic melanoma patients. However, most responses are incomplete (due to innate and adaptive drug resistance) and, among those patients with objective tumour responses, the median duration of response is ~6 months due to acquired drug resistance^16^,^17^. RAF inhibitor resistance can be achieved through several mechanisms, including amplification or mutations in upstream kinases (RAFs, MEK1 or COT kinases or genetic alteration in upstream activators such as NRAS, KRAS or epidermal growth factor receptor), ultimately leading to reactivation of the MEK/ERK pathway in a majority of cases^18^,^19^,^20^,^21^,^22^,^23^,^24^,^25^. Other mechanisms of resistance have also been identified, including activation of the PI3K (phosphoinositide 3-kinase)/AKT pathway^23^,^26^,^27^. Thus, there is an intense effort to further understand mechanisms of innate, adaptive and acquired resistance. Here we describe that MLK1–4 directly phosphorylate MEK and activate the MEK/ERK pathway independently of RAF kinases. Moreover, we find that increased expression of MLKs correlates with drug resistance in patients, implicating their potential role as mediators of resistance to RAF inhibitors in melanoma.

## Results

### MLKs are direct MEK kinases that activate the ERK pathway

**Figure 1:**
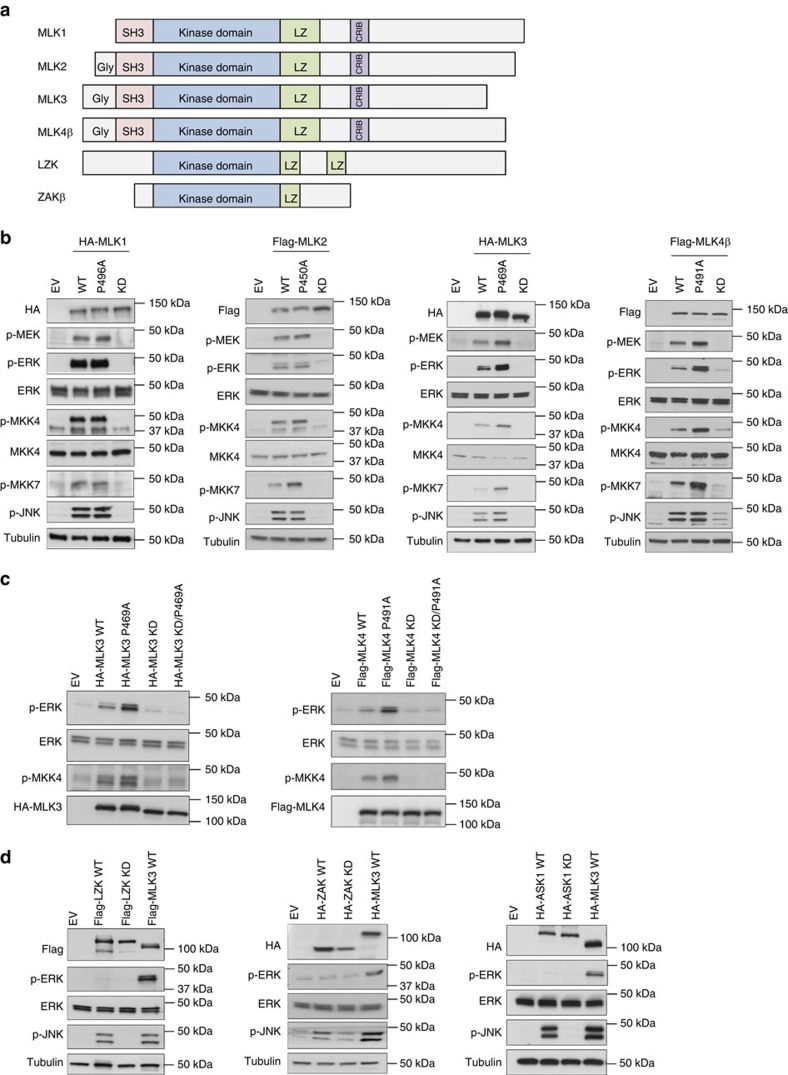
MLK1–4 are upstream regulators of the ERK pathway. (**a**) Schematic representation of MLKs domains (SH3, Src homology-3 domain; LZ, leucine-zipper; CRIB, Cdc42/Rac-interactive binding region). (**b**) HEK293T cells were transiently transfected with Flag- or HA-tagged MLK1–4 WT (wild type); P496A–MLK1, P450A–MLK2, P469A–MLK3 and P491A–MLK4β; KD (kinase dead) (D294A–MLK1 (DFG motif), K125M–MLK2, K144M–MLK3 and K151M–MLK4β), or EV (empty vector) control. After 48 h, cells were lysed and analysed by western blotting. (**c**) Activation of the MEK/ERK pathway is dependent on MLK kinase activity. HEK293T cells were transiently transfected with HA-MLK3 or Flag-MLK4β constructs or EV. Cell lysates were analysed by western blotting. (**d**) LZK, ZAKβ and ASK1 activate JNK but not MEK/ERK. HEK293T cells were transiently transfected with EV or Flag-LZK WT or KD (K195M), HA-ZAKβ WT or KD (K45M), HA-ASK1 WT or KD (K709M). After 48 h, cells were lysed and analysed by western blotting. HA- or Flag-tagged MLK3 WT is a positive control. All results are representative of three independent experiments.

In an effort to evaluate the role of the mixed lineage family of kinases (Fig. 1a) in regulating downstream signalling pathways, we overexpressed WT (wild type), KD (kinase dead) and constitutively active MLK1***–***4 in HEK293T cells and assessed mitogen-activated protein kinase (MAPK) pathway activation. Exogenous expression of MLK1***–***4 resulted in activation of MKK4, MKK7 and the JNK pathway (Fig. 1b). We also observed that the MLKs are robust activators of the MEK/ERK pathway and this requires a catalytically competent kinase domain (Fig. 1b). Activation of the MEK/ERK pathway was enhanced by expression of a constitutively active MLK3 and MLK4β (Fig. 1b), where a proline residue is mutated to an alanine (referred to as P469A for MLK3 and P491A for MLK4β hereafter). This mutation results in a constitutively active MLK mutant, by relieving autoinhibitory binding of this conserved proline to the SH3 domain^1^. Homologous mutations in MLK1 and MLK2 did not increase pathway activation, indicating that MLK1/2 are regulated in a unique manner and autoinhibition may occur by binding to other carboxy-terminal regions of the kinases (Fig. 1b). To confirm that activation of the MEK/ERK pathway was dependent on MLK kinase activity, rather than scaffolding effects, we generated constitutively active KD mutants for MLK3 and MLK4 that would serve as an open scaffold, but lack kinase activity (KD/P469A or KD/P491A, respectively). Both mutants were unable to activate the MEK/ERK pathway (Fig. 1c), indicating a functional kinase domain is required for activation of the ERK pathway. Evaluation of other distant MLK family members and another MAP3K indicated that this function is specific to MLK1–4, as exogenous expression of LZK, ZAKβ or ASK1 did not alter signalling through the MEK/ERK pathway (Fig. 1d). This implies that regulatory domains present on MLK1–4, such as the SH3 domain or the CRIB domain, are required for regulation of the MEK/ERK pathway.

**Figure 2:**
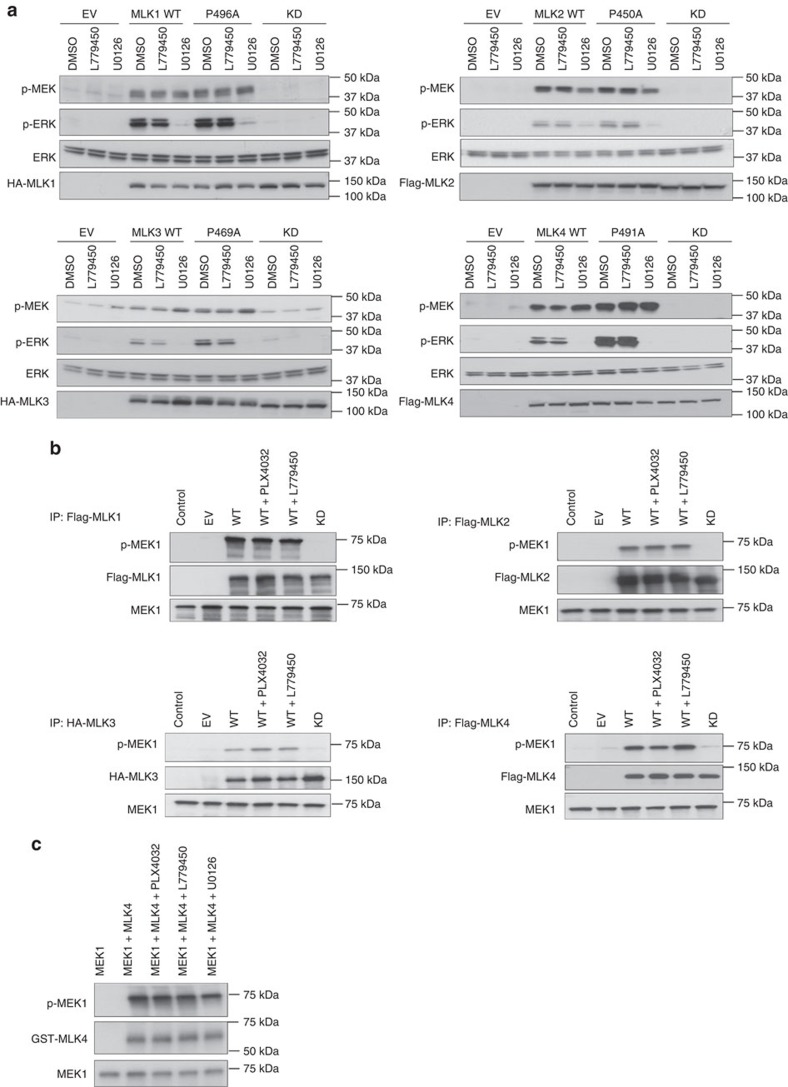
MLK1–4 phosphorylate MEK in the presence of RAF inhibitors. (**a**) HEK293T cells were transiently transfected with Flag- or HA-tagged MLK1–4 constructs or EV (empty vector) control. Cells were treated with 5 μM L779450, 5 μM U0126 or DMSO for 1 h and lysed. Cell lysates were analysed by western blotting. (**b**) MLK1–4 are MEK1 kinases. Flag- or HA-tagged MLK1–4 WT, KD or EV control were transiently transfected in HEK293T cells. Forty-eight hours later, cells were treated with 1 μM PLX4032 (vemurafenib), 5 μM L779450 or DMSO for 1 h before lysis. MLK1–4 were immunoprecipitated and subjected to a kinase assay with kinase-inactive MEK1. Control sample does not include any cell lysate. (**c**) Kinase-inactive MEK1 and purified GST-MLK4β kinase domain (isolated from baculovirus-infected insect cells) were subjected to *in vitro* kinase assay in the presence or absence of inhibitors: 1 μM PLX4032 (vemurafenib), 5 μM L779450 or 5 μM U0126. All results are representative of three independent experiments.

To determine the mechanism used by MLK1–4 to activate the MEK/ERK pathway, we overexpressed the MLKs in HEK293T cells and treated the cells with a pan-RAF inhibitor (L779450) or a MEK inhibitor (U0126). MLK1–4 activated the MEK/ERK pathway in the presence of the RAF inhibitor but not the MEK inhibitor, indicating that MLKs directly phosphorylate MEK (Fig. 2a and Supplementary Fig. 1a in H157 cells; note both L779450 and U0126 blocked EGF-stimulated ERK activation in cells, Supplementary Fig. 1b). These results were verified with additional RAF and MEK inhibitors, sorafenib and AZD6244, respectively (Supplementary Fig. 1c,d). To assess whether the MLKs directly phosphorylate MEK, we performed *in vitro* kinase assays using purified inactive MEK1. Immunoprecipitated full-length MLK1–4 directly phosphorylated KD MEK1 and the activity of the kinases was not altered by the presence of RAF or MEK inhibitors (Fig. 2b and Supplementary Fig. 1e). To rule out the possibility that other kinases could co-precipitate with MLKs and phosphorylate MEK1, we used purified GST-MLK4 kinase domain in an *in vitro* kinase assay and observed that the MLK4 kinase domain directly phosphorylated MEK1 and was not inhibited by RAF or MEK inhibitors (Fig. 2c). This is consistent with our previous report that purified GST-MLK1 kinase domain can directly phosphorylate KD MEK1 *in vitro*^28^.

### MLKs reactivate the ERK pathway in melanoma cells

**Figure 3:**
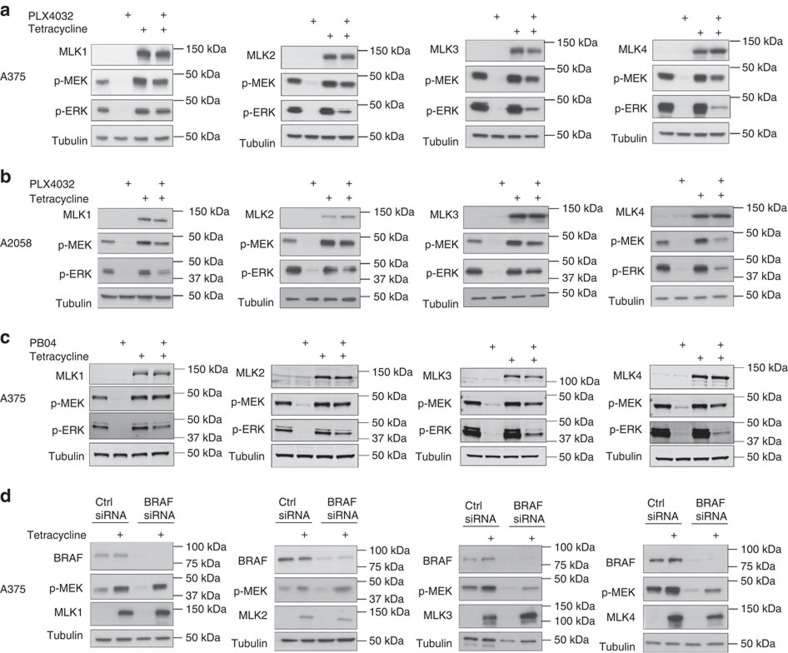
MLK1–4 reactivate the MEK/ERK pathway in V600E-positive melanoma cells independently of the RAF kinases. (**a**,**b**) Expression of MLKs in A375 and A2058 cell lines was induced by addition of tetracycline. After 24 h, cells were treated with 1 μM PLX4032 (vemurafenib) or DMSO for 1 h, lysed and analysed by western blotting. (**c**) Expression of MLK1–4 in A375 cells was induced by addition of tetracycline. After 24 h, cells were treated with 1 μM PB04 or DMSO for 1 h, lysed and analysed by western blotting. (**d**) Expression of MLKs reverses the effect of BRAF knockdown on activation of the MEK/ERK pathway. A375 cells were transfected with siRNA against BRAF or control siRNA. After 24 h, expression of MLKs was induced by tetracycline. Cells were lysed and analysed by western blotting. All results are representative of three independent experiments.

Based on our proposed mechanism whereby MLKs can activate the MEK/ERK pathway in a manner independent of the RAF kinases, we sought to determine whether MLKs may mediate reactivation of this pathway in the presence of RAF inhibitors in V600E-positive melanoma cells. We transiently expressed MLK1–4 and their respective KD mutants in A375 cells and treated the cells with vemurafenib (PLX4032). We observed that expression of MLKs reactivated the MEK/ERK pathway in the presence of vemurafenib in a kinase-dependent manner (Supplementary Fig. 2a). Next, we generated melanoma cell lines (both with V600E mutations: A375 and A2058) where MLK expression could be induced in response to tetracycline. Vemurafenib effectively inhibited phosphorylation of MEK and ERK in these melanoma cell lines, while induced expression of MLK1–4 promoted reactivation of the MEK/ERK pathway despite the presence of vemurafenib (Fig. 3a,b). Treatment of cell lines with MEK inhibitors prevented phosphorylation of the pathway even with the expression of MLKs, confirming that the MLKs directly activate MEK (Supplementary Fig. 2b). To further validate that MLK1–4 activate the MEK/ERK pathway independently of RAF kinases we used PB04, a non-paradox-inducing RAF inhibitor that does not promote transactivation of RAF isoforms^29^. Expression of MLK1–4 reactivated the MEK/ERK pathway in the presence of PB04 in the A375 and A2058 melanoma cell lines, confirming that MLKs directly phosphorylate MEK rather than act as a scaffold to promote RAF-mediated activation of the MEK/ERK pathway (Fig. 3c and Supplementary Fig. 2c).

To assess whether the V600E mutant allele of BRAF was required for MEK phosphorylation by the MLKs in melanoma cells, we depleted A375 cells of BRAF using small interfering RNA (siRNA) and observed that MLK1–4 induction was still able to activate the MEK/ERK pathway, indicating that the BRAF^V600E^ allele is dispensable for MLK-mediated activation of MEK in these cells (Fig. 3d). Consistent with these being RAF-independent effects, the MLKs could promote activation of the MEK/ERK pathway in the presence of the pan-RAF inhibitor (L779450), ruling out the possibility that MLKs mediate their effects through ARAF or CRAF (Supplementary Fig. 2d,e).

**Figure 4:**
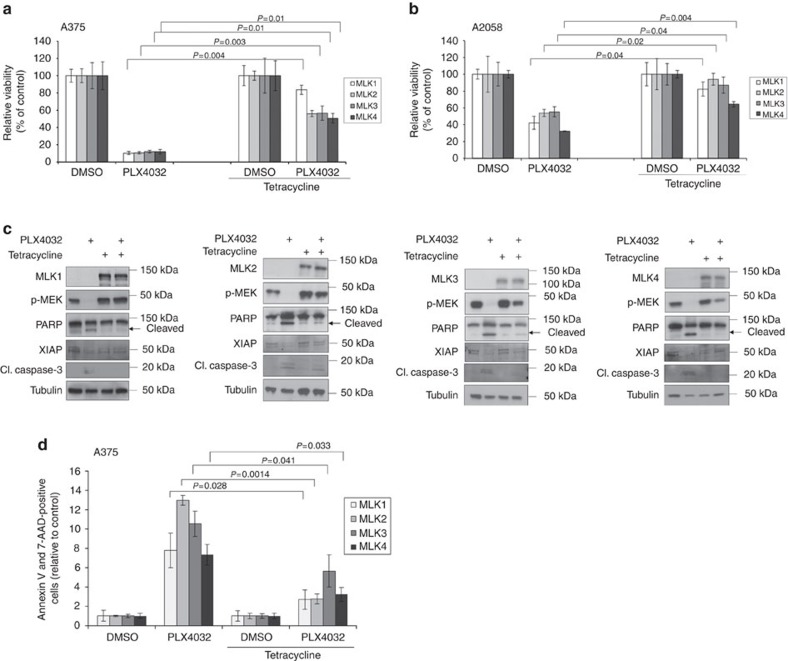
Induction of MLK1–4 promotes survival of vemurafenib-treated cells. (**a**,**b**) Expression of MLKs in A375 and A2058 cell lines was induced by tetracycline followed by treatment with 1 μM PLX4032 (vemurafenib) or DMSO for 4 days. Viability was determined by MTT assay. Error bars indicate ±s.e.m. from three independent experiments performed in triplicate (*n*=9). *P*-values calculated by Student’s *t*-test. (**c**) Expression of MLKs in A375 cells was induced by tetracycline followed by treatment with vemurafenib for 2 days. After that time, cells were lysed and analysed by western blotting. Cleaved PARP is marked with an arrow. All results are representative of three independent experiments. (**d**) Expression of MLK1–4 in A375 cells was induced by tetracycline followed by treatment with 1 μM PLX4032 (vemurafenib) or DMSO for 2 days. Apoptosis was assessed by staining for annexin V and 7-AAD, and analysed by Muse Cell Analyser. Error bars indicate ±s.e.m. from at least three independent experiments performed in duplicate (*n*=6). *P*-values calculated by Student’s *t*-test.

To determine whether MLKs can promote resistance to vemurafenib in terms of cell survival, we assessed cell viability by MTT (3-(4,5-dimethylthiazol-2-yl)-2,5-diphenyltetrazolium bromide) assay. Importantly, expression of each MLK isoform significantly increased survival in melanoma cells treated with vemurafenib (Fig. 4a,b). The reactivation of the MEK/ERK pathway by expression of MLKs is complete for MLK1 in A375 cells and MLK3 in A2058 cells, whereas MEK/ERK phosphorylation is only partially restored by MLK2–4 in A375 cells and MLK1, MLK2 and MLK4 in A2058 cells (Fig. 3a,b). Nevertheless, this level of pathway reactivation is sufficient to promote MLK-mediated survival in the presence of vemurafenib in both melanoma cell lines (Fig. 4a,b). To verify the survival advantage conferred by the MLKs in the presence of vemurafenib, we monitored poly (ADP-ribose) polymerase (PARP) and caspase-3 cleavage, indicators of apoptosis, and we assessed the level of XIAP, a member of the inhibitors of apoptosis family of proteins. Induced expression of all four MLKs abrogated the reduction in XIAP levels, as well as the cleavage of PARP and caspase-3, in response to vemurafenib in the A375 cells, suggesting that the MLKs can mediate resistance to vemurafenib-induced apoptosis (Fig. 4c). Lastly, we verified the MLK-mediated resistance to vemurafenib using an annexin V assay and found that significantly fewer cells underwent apoptosis when MLKs were expressed (Fig. 4d).

To investigate whether activation of the JNK pathway is important for the survival effects mediated by MLKs, we treated melanoma cells with vemurafenib and a JNK inhibitor (SP600125). Addition of SP600125 effectively inhibited MLK-induced activation of the JNK pathway (monitored by decreases in c-Jun phosphorylation), but did not affect resistance to vemurafenib mediated by MLK1, 2 or 4 (Supplementary Fig. 3a,b). For MLK3, treatment of cells with the JNK inhibitor dramatically decreased the expression of MLK3 and the resistance to vemurafenib mediated by MLK3 (Supplementary Fig. 3a,b).

### MLK upregulation correlates with drug resistance in patients

**Figure 5:**
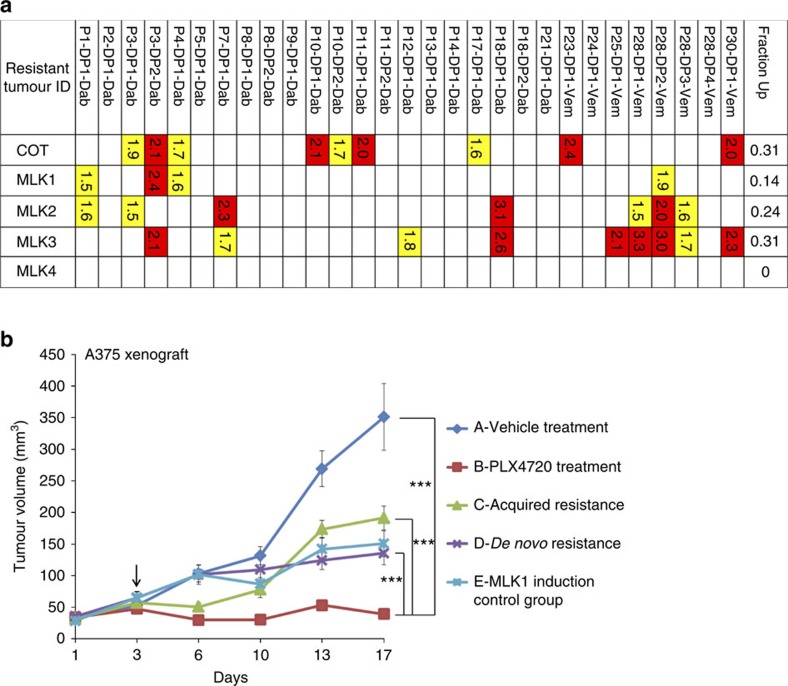
MLK alterations correlate with drug resistance in melanoma patients and in a mouse model. (**a**) The recurrence of MLK1–4 upregulation in a set of 29 disease progression biopsies from 21 patients (along with COT kinase expressions included for reference). The *x*-axis lists the disease progression tumour IDs annotated with their BRAF inhibitor treatment. P, Patient, DP, Disease Progression, Dab, Dabrafenib, Vem, Vemurafenib. The RNAseq data were downloaded from the set published by Rizos *et al.*^30^ Tiles with colour shading signify upregulation of the gene expression (fold-change≥2 (red) or 1.5≤fold-change≤2 (yellow)) in the DP samples as compared with their respective baselines. (**b**) Induced expression of MLK1 promotes tumour survival in a xenograft mouse model. A375 cells were engrafted in SCID mice. After tumour establishment, mice were treated with vehicle (groups A and E) or PLX4720 (45 mg kg^−1^) (group B, C and D). The first day of the treatment with PLX4720 is marked with an arrow (day 3). MLK1 expression by doxycycline induction started 2 days before the first treatment for groups D and E (day 1), or 5 days after the first treatment for group C (day 8). Mean tumour volumes ±s.e.m. are shown (*n*=10 mice per group), ***P<0.001 by Student’s *t*-test.

To assess whether alterations in MLKs correlated with resistance to RAF inhibitor therapy, we analysed mRNA expression of MLK1–4 from the data set recently published by Rizos *et al.*^30^ The authors performed gene expression analysis on matched pretreatment and disease progression biopsies from 21 metastatic melanoma patients^30^. All patients were BRAF^V600^-positive and treated with RAF kinase inhibitors, vemurafenib or dabrafenib^30^. Here we evaluated mRNA expression of MLK1–4 before and during disease progression in 29 pair-wise comparisons in 21 patients (Fig. 5a). We detected upregulation of MLK1–3 in 9 of 21 (42.9%) patients, or in 12 of 29 (41.4%) disease progressive tumours (versus their patient-matched baseline; Fig. 5a). Multiple MLKs were upregulated at the sites of disease progression with respect to their baseline melanoma in five patients, whereas a single MLK was upregulated in four patients (Fig. 5a). Ten disease progression sites (out of 12) that displayed enhanced expression of MLK1-3 showed reactivation of the MAPK pathway (Supplementary Table 1)^30^. In addition, five disease progression sites lacked other known mechanisms of acquired BRAF inhibitor resistance (Supplementary Table 1)^30^. Three melanoma patients displayed upregulation of MLKs together with COT kinase upregulation (Fig. 5a). COT kinase upregulation has previously been described as a mechanism of resistance to RAF inhibitors in melanoma^31^; thus, we used it as a positive control reference in our study. We noted that the ranges of upregulation of MLK1–3 (1.5- to 3.3-fold increase) are similar to that of COT kinase (1.6- to 2.4-fold increase), strongly indicating that the upregulation events that we observe are biologically relevant (Fig. 5a). In summary, these clinical data suggest that MLKs are used to promote resistance to RAF inhibitor therapy in patients.

### Expression of MLKs promotes resistance in mouse models

To assess whether MLKs could promote resistance in a xenograft mouse model, we injected mice with A375 cells that had stably incorporated, doxycycline-inducible MLK1. All mice formed engrafted tumours within 18 days, at which point we commenced treatment with either vehicle control or PLX4720 (a vemurafenib analogue). Expression of MLK1 was induced 5 days after the start of PLX4720 treatment to assess acquired resistance (group C) or 2 days before the first treatment with the drug to evaluate *de novo* resistance (group D) (Fig. 5b). PLX4720 treatment significantly suppressed tumour growth (group B), while MLK1 induction decreased the overall tumour response to PLX4720 in both models of resistance, acquired and *de novo* (Fig. 5b). Group C, representing acquired resistance, showed initial response to PLX4720 treatment until MLK1 expression was induced by administration of doxycycline on day 8, and following doxycycline treatment the tumours started gradually increasing in size (Fig. 5b). Group D, representing *de novo* resistance, did not show initial response to PLX4720 due to MLK1 expression before the start of the treatment (Fig. 5b). Analogous results were observed with mouse xenografts using MLK3-inducible A375 cells (Supplementary Fig. 4). This indicates that increased expression of MLKs can promote resistance to RAF inhibitors *in vivo*, which is consistent with the observation that increased expression of MLKs correlates with resistance at sites of disease progression in patients (Fig. 5a). We also observed that MLK1 or MLK3 induction alone (group E) reduced tumour growth compared with the vehicle-treated group (group A) (Fig. 5b and Supplementary Fig. 4). This is likely to be due to sustained activation of the JNK pathway, which can promote cell death, and in the absence of BRAF^V600E^ inhibition the JNK pathway will be the primary pathway activated downstream of overexpressed MLK1 or MLK3. Our data indicate that the MLKs will promote melanoma cell survival when BRAF^V600E^ is inhibited and melanoma cells seek other mechanisms to reactivate the MEK/ERK pathway.

### MLK2 promotes resistance to vemurafenib in cell line model

**Figure 6:**
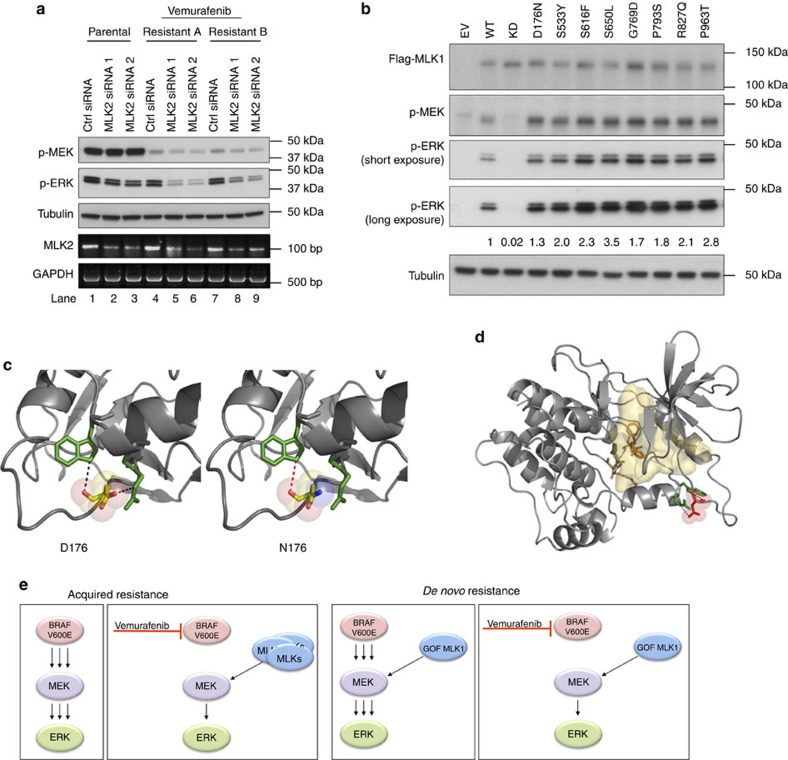
MLKs promote resistance to vemurafenib. (**a**) MDA-MB-435 parental and resistant clones A and B were transfected with siRNA against MLK2 or control siRNA and lysed after 48 h. Resistant A and B were grown in the presence of vemurafenib. Activation of the MEK/ERK pathway was analysed by western blotting and the level of MLK2 was verified by RT–PCR. All results are representative of three independent experiments. (**b**) The presence of GOF mutations in MLK1 in melanoma patients. H157 cells were transiently transfected with EV (empty vector) control, MLK1 WT, KD (kinase dead) or MLK1 mutants. Cell lysates were analysed by western blotting. Results are representative of three independent experiments. Densitometry was performed using ImageJ. P-ERK was normalized to tubulin and Flag expression levels, and compared directly with WT MLK1. (**c**) Comparison of the structural changes occurring following mutation at site D176. The WT D176 residue forms two polar contacts between W301 and R303 (shown in green), whereas mutant N176 is only able to form one polar contact to W301 (atoms at mutation site are coloured as follows: carbon–yellow; oxygen–red; nitrogen–blue). (**d**) Structure of the MLK1 kinase domain highlighting the position of the D176 mutation site (red) with respect to the position of the ATP-binding pocket (shaded yellow). DFG and HxD-motifs are highlighted in orange and mustard, respectively; residues taking part in polar contacts with the D176 residue are shown in green; images were created using PyMol v1.3. (**e**) Model depicting MLKs can directly phosphorylate MEK to mediate resistance to RAF inhibitors in melanoma tumours harbouring V600E mutation; acquired resistance mediated by upregulation of MLKs and *de novo* resistance driven by GOF mutations in MLK1.

To test whether endogenous MLKs mediate resistance, we established two drug-resistant V600E-positive melanoma cell lines, MDA-MB-435 (ref. 32) and A375, by chronic treatment with 1 μM vemurafenib. These cells were maintained in the presence of the drug and showed sustained phosphorylation of ERK when compared with the parental cell line (Fig. 6a, lane 1, 4, 7 and Supplementary Fig. 5a). To determine whether MLKs were mediating resistance, we used siRNA to deplete MLK1–4 in both resistant cell lines. We observed that depletion of MLK2, but not MLK1, 3 or 4, in MDA-MB-435 cells led to decreased MEK/ERK pathway activation (Fig. 6a and Supplementary Fig. 5b). Interestingly, MLK2 depletion modestly inhibited MEK phosphorylation and robustly inhibited phosphorylation of the downstream target ERK, which probably reflects the presence of more active ERK phosphatases compared with MEK phosphatases (Fig. 6a). We were unable to detect endogenous MLK2 by western blotting due to low-quality antibodies; thus, knockdown was assessed by reverse transcription–PCR (RT–PCR) (Fig. 6a). Suppression of ERK pathway activation due to MLK2 depletion correlated with a reduction in cell viability in resistant MDA-MB-435 cells (Supplementary Fig. 5c). Depletion of MLKs from drug-resistant A375 cells did not alter MEK/ERK pathway activation, suggesting that these cells use different mechanisms to reactivate ERK (Supplementary Fig. 5d). This result is in line with previous reports of alternative resistance mechanisms described in the A375 cell line^21^.

### Gain-of-function mutations in MLK1 in melanoma patients

*MAP3K9* (MLK1) has been identified as a gene that is frequently mutated in melanoma^8^. Fourteen per cent (12 of 85) of melanoma patients evaluated by this study had MLK1 mutations, including 6% (5 of 85) of patients that had both BRAF^V600E^ and MLK1 mutations^8^. The authors characterized a truncation mutation, W333*, as a loss-of-function mutation, and it was proposed that MLK1 acts as a tumour suppressor in melanoma; however, this mutation was not confirmed in the primary tumour sample^8^. Here we determined the functional consequences of other MLK1 mutations from melanoma patients that were identified by Stark *et al.*^8^ We observed that several of these mutations (including recurrent mutations) were gain-of-function (GOF) towards the MEK/ERK pathway when compared with WT MLK1 (Fig. 6b). In total, 10.6% (9/85) of melanoma patients have GOF mutations in MLK1; however, some mutations were neutral or loss-of-function (Supplementary Fig. 5e). To provide insight into the molecular mechanism that causes the D176N mutation to activate MLK1, we performed structural analysis, and based on a comparison between the homology modelled D176N mutant and the MLK1 crystal structure, we observe the change from Asp to Asn results in loss of a polar contact, specifically the Arg at position 303 (Fig. 6c). Loss of this polar contact between Asp 176 and Arg 303 could cause destabilization, allowing the ATP binding site to be more open and shifting the equilibrium of the kinase into a more active conformation (Fig. 6d). The presence of GOF mutations in MLK1 in V600E-positive patients suggests these patients could be predisposed to *de novo* resistance. These data emphasize the importance of MLK1 in melanoma as a potential drug target.

## Discussion

Our data demonstrate that MLK1–4 are MEK kinases that are able to directly phosphorylate MEK to activate the MEK/ERK pathway independently of the RAF kinases. These data provide a new layer of complexity in regards to MAPK signalling; we have shown that the MLKs are not only able to phosphorylate MKK4/7 to regulate the JNK pathway, but also MEK1/2 to regulate the ERK pathway. It was previously reported that MLK3 acts as a scaffold for BRAF to activate the MEK/ERK pathway; however, our data demonstrate that the MLKs act directly at the level of MEK, and through phosphorylation of MEK can activate ERK. Interestingly, we only observed this for MLK1–4, while less related MLK family members such as LZK and ZAK were unable to activate the MEK/ERK pathway on overexpression. This suggests that domains specific to MLK1–4, such as the SH3 domain, are likely to be important in mediating scaffolding interactions that will dictate MLK regulation of the ERK pathway compared with the JNK pathway. It will be important to fully understand what dictates pathway specification by this understudied family of kinases. Much remains to be determined regarding the nature of when and how the MLKs regulate the MEK/ERK pathway and future studies should reveal exciting revelations regarding the molecular mechanisms important for differential regulation of downstream components.

From our observation that the MLKs could phosphorylate MEK, we then aimed to determine whether MLKs could facilitate resistance to vemurafenib. Resistance generally occurs through reactivation of the MEK/ERK pathway, although activation of the PI3K/AKT pathway by receptor tyrosine kinase upregulation^18^,^19^,^20^,^21^,^22^,^23^,^24^ or by genetic mechanisms contributes to MAPK-independent survival^18^,^19^,^20^,^21^,^22^,^23^,^24^,^27^. Reactivation of the MEK/ERK pathway can be achieved by increased expression or mutational activation of the RAF kinases, MEK1, or upstream activators (such as NRAS, KRAS, platelet-derived growth factor receptor, epidermal growth factor receptor or MET-ligand induced activation)^20^,^24^,^25^,^33^. Expression of the p61 BRAF^V600E^ splice variant shows enhanced dimerization rendering a subset of melanoma patients resistant to vemurafenib^19^,^34^. In addition, kinases that act independently of RAF to phosphorylate MEK have the potential to mediate resistance, such as COT kinase^31^,^33^. Our study identifies an additional novel mechanism whereby MLK1–4 can reactivate the MEK/ERK pathway independently of RAFs (Fig. 6e). We have shown that expression of MLKs promotes resistance to RAF inhibitors (including vemurafenib) via direct MEK phosphorylation in melanoma cell lines, in mouse models of resistance and in a cell line model of resistance. Importantly, increased expression of MLK1-3 occurs at sites of disease progression across patients treated with RAF inhibitors, further confirming the role of MLKs in mediating resistance to RAF inhibitor therapy. Upregulation of MLKs was detected in several drug-resistant tumours that lack other known mechanisms of acquired resistance (Supplementary Table 1)^30^, implying that increased expression of MLKs is sufficient to reactivate the MAPK pathway in those tumours. On the other hand, a subset of drug-resistant tumours display upregulation of MLKs together with expression of BRAF splice variants (Supplementary Table 1)^30^, a previously described mechanism of resistance^19^,^34^. This suggests that multiple mechanisms might contribute to reactivation of the MAPK pathway and tumour survival. Alternatively, the upregulation of MLKs may have a transient effect on MAPK pathway reactivation until further mechanisms of drug resistance are developed. It is currently unclear how early MLK overexpression occurs subsequent to the initiation of RAF-targeted therapies. Delineation of such temporal expression dynamics may provide additional insight into the roles of these MEK kinases in early-adaptive versus late-acquired resistance (explaining incomplete initial responses versus subsequent clinical relapses).

Finally, we have characterized several MLK1 mutations observed in melanoma patients to be GOF mutations. The presence of intermediate-activating mutations in MLK1 suggests that MLK1 mutations could predispose V600E-positive patients to *de novo* resistance (Fig. 6e). Further studies will be aimed at determining whether GOF mutants of MLK1 can enhance melanomagenesis, suggesting MLK1 may be a melanoma oncogene. In conclusion, elucidating mechanisms of resistance to RAF inhibitors in melanoma patients is essential to achieve long-term progression-free survival and further understand the basic biology of this disease. Here we have identified the MLKs as a family of kinases that can directly phosphorylate MEK to mediate resistance to RAF inhibitors.

## Methods

### Cell lines and reagents

HEK293T and H157 cells were cultured in DMEM supplemented with 10% FCS, 1% penicillin/streptomycin and 2 mM L-glutamine. A375, A2058 and MDA-MB-435 cells were cultured in RPMI supplemented with 10% FCS, 1% penicillin/streptomycin and 2 mM L-glutamine. The H157 non-small-cell lung carcinoma and A2058 cell lines were kind gifts from Dr Phillip Dennis and Dr Geoff Margison. All other cell lines were obtained from ATCC. PLX4032 (vemurafenib), AZD6244 and sorafenib were purchased from Selleck Chemicals. L779450 and SP600125 were obtained from Merck. U0126 was purchased from Cell Signaling Technology. PB04 was provided by Dr Gideon Bollag (Plexxikon Inc.). All inhibitors were dissolved in dimethyl sulphoxide (DMSO) and stored at −20 °C.

### Generation of tetracycline-inducible cell lines

Parental A375 and A2058 melanoma cell lines were used to generate cells with tetracycline-inducible expression of MLKs. MLK1–4 plasmids (cloned into pLenti/TO/V5-DEST vector) and pLenti3.3/TR (for tetracycline repressor expression) were transfected into 293FT cells using Lipofectamine2000 to generate lentiviral stock. Cells were transduced with lentiviral stocks and cell lines generated by antibiotic selection (Blasticidin (Invitrogen) and Geneticin (Gibco)). Tetracycline (Invitrogen) was used to induce expression of MLKs.

### Generation of vemurafenib-resistant cell lines

The MDA-MB-435 and A375 drug-resistant cell lines were generated by treating parental cells with 1 μM vemurafenib. After about 1 month, colonies of resistant cells were trypsinized, picked into 12-well plates and expanded as necessary on reaching confluency. At each stage, medium was removed daily and replaced with fresh medium containing vemurafenib. For all experiments, 1 μM vemurafenib was added to resistant cells when seeding and again after 24 h where appropriate.

### Protein lysate preparation and immunoblots

Forty-eight hours after transient transfection, cells were treated with inhibitors or DMSO vehicle control for the appropriate time before lysis with Triton X-100 lysis buffer (Cell Signaling Technology) supplemented with protease inhibitor tablet (Roche). Primary antibodies: p-ERK1/2 (T202/Y204), p-MEK (S217/221), ERK1/2, MEK, p-MKK4 (S257/T261), p-MKK7 (S271/T275), MKK4, p-JNK (T183/Y185), JNK, MLK1, PARP, XIAP, cleaved caspase-3 (Cell Signaling Technology), MLK2, MLK3 (Epitomics), MLK4 (Bethyl), HA (Covance), Tubulin, Flag M2 (Sigma) and BRAF (Santa Cruz). Tubulin antibody was used at 1:10,000 dilution and Flag M2 at 1:5,000 dilution. All other antibodies were used at 1:1,000 dilution. Uncropped scans of the most important immunoblots are shown in Supplementary Fig. 6.

### Plasmids and transfections

MLK1 in entry vector was purchased from OriGene. MLK2, 3 and 4β were obtained from GeneCopoeia as shuttle clones. MLKs were cloned into Flag- or HA-tagged destination vectors. ASK1 and ZAKβ (Addgene) were cloned into Flag- or HA-tagged destination vectors. LZK complementary DNA was prepared from RNA extracted from HEK293T cells, *attB* flanking regions were added by PCR and the BP clonase reaction used to insert LZK into pDONR221. Mutations were introduced using a Site-Directed Mutagenesis Kit (Stratagene). HEK293T and H157 cells were transiently transfected using Attractene (Qiagen) according to the manufacturer’s protocol. A375 cells were transfected using Lipofectamine2000 (Invitrogen) according to the manufacturer’s protocol. DharmaFECT1 (Thermo Scientific) was used for siRNA transfections. siRNA against BRAF (5′-AAA GAA UUG GAU CUG GAU CAU-3′) was obtained from Thermo Scientific. MLK2 siRNA were purchased from Invitrogen (5′-GGC UUU GAG CAU AAG AUC A-3′) and Santa Cruz (sc-39111). siRNA against MLK1 were obtained from OriGene (5′-ACCUUUGAAUAGAGUGUUAUCUGGG-3′) and Ambion (5′-GACCAUCUUUCACGAAUAU-3′), siRNA against MLK3 were obtained from Thermo Scientific (J-003577-11) and Ambion (5′-CGUGAUCUCAAGUCCAACA-3′), siRNA against MLK4 were obtained from Thermo Scientific (5′-GGAAAGAUGCUCAGAGAGAUU-3′ and 5′-AGGAGAAGCCCAAGGUAAAUU-3′).

### Immunoprecipitation and kinase assays

Cells were seeded into six-well plates and transiently transfected the next day. Forty-eight hours later, cells were treated with inhibitors or DMSO and lysed with Triton X-100 lysis buffer (Cell Signaling Technology). Cell lysates were incubated with anti-Flag M2 affinity gel (Sigma) for 2 h or rabbit HA antibody (Cell Signaling Technology) diluted 1:50 for 2 h followed by incubation with protein A/G beads (Thermo Scientific) for 1 h. Beads were washed with lysis buffer and kinase buffer (Cell Signaling Technology), and kinase assay was performed in the presence of 200 μM ATP and kinase-inactive MEK1 purified from baculovirus-infected insect cells (Carna Biosciences), at 30 °C for 30 min. Following addition of 4 × reduced SDS sample buffer, proteins were resolved by SDS–PAGE and analysed by western blotting.

### *In vitro* kinase assay

Human glutathione *S*-transferase (GST)-tagged MLK4 kinase domain expressed using baculovirus expression system (Carna Biosciences) was incubated with kinase-inactive MEK1 (Carna Biosciences) in the absence or presence of specific inhibitors. Kinase assay was performed with 200 μM ATP at 30 °C for 30 min. Following addition of 4 × reduced SDS sample buffer, proteins were resolved by SDS–PAGE and analysed by western blotting.

### Cell proliferation and viability assays

Cells were seeded into 96-well plates and tetracycline was added into the medium the following day. After 24 h, cells were treated with inhibitors for 4 days. MTT Kit (Sigma) was used according to the manufacturer’s instructions.

### Annexin V and dead cell assay

Cells were seeded into 12-well plates and tetracycline was added the following day. After 24 h, cells were treated with PLX4032 for 2 days. Annexin V and Dead Cell Kit (Muse Annexin V and Dead Cell Reagent) was used according to the manufacturer’s instructions and cells were analysed by Muse Cell Analyzer.

### Mouse xenografts and *in vivo* drug studies

All procedures involving animals were approved by CRUK Manchester Institute’s Animal Welfare and Ethical Review body in accordance with the Animals (Scientific Procedures) Act 1986 and according to the guidelines of the Committee of the National Cancer Research Institute^35^. Five- to six-week-old female nude mice were injected subcutaneously with 5 × 10^6^ MLK1-inducible A375 cells (ten mice per group). For MLK3-inducible model, 5 × 10^6^ A375 cells were injected subcutaneously into 8- to 10-week-old female nude mice (three mice per group). Tumours were allowed to establish, size matched and then the mice were randomly allocated to groups of ten animals (three for MLK3 model). No blinding was used in the treatment schedules for these studies. Based on literature precedents, groups of ten animals were used to provide sufficient animals per cohort, to provide statistically significant data, although keeping the number of animals used to a minimum. For MLK1 xenografts, treatment was by oral gavage daily with vehicle (5% DMSO, 95% water) or PLX4720 45 mpk and/or doxycycline. Tumour size was determined by caliper measurements of tumour length, width and depth, and volume was calculated as volume=0.5236 × length × width × depth (mm^3^). For MLK3 xenografts, vemurafenib treatment was administered by daily gavage at a dose of 25 mpk. Doxycycline diet started on the same day as vemurafenib treatment or 5 days later. Tumour formation was monitored every few days and tumour volume based on caliper measurements was calculated by the formula: tumour volume=(length × width × width)/2. Mice for both experiments were culled when tumours reached max-permitted volume 1,200 mm^3^ or after 5 weeks post injection of A375 cells.

### Microarray data

The mRNA expressions BRAFi samples are taken from the microarray data set published by Rizos *et al.*^30^ (accession number GSE50509). They obtained the total RNA from fresh-frozen melanoma samples from patients before dabrafenib (Dab) or vemurafenib (Vem), and at the time of disease progression (DP). The expression data was done on Illumina HumanHT-12 V4.0 Expression BeadChip microarray, which we normalized using the *normaliseIllumina* function in the *beadarray* R package. Significant expression alterations are defined by 50% up- or downregulation in line with the earlier report of at least 1.5-fold upregulation of COT kinase in two out of three BRAF inhibitor-treated patient samples^31^. In the subset of patients with replicate data (P7-Pre, P8-Pre, P10-Pre, P11-Pre, P11-DP1-Dab, P11-DP2-Dab, P13-Pre, P18-Pre and P24-Pre), the fold change is computed as the fold change of the median expression value across the replicates.

### Reverse transcription–PCR

RT***–***PCR was used to assess expression and siRNA knockdown of MLK2 in resistant MDA-MB-435 cell lines. An RNeasy Mini Kit (Qiagen) was used to extract RNA. RT–PCR was performed using a OneStep RT–PCR Kit (Qiagen) according to the manufacturer’s protocol. The PCR conditions were as follows: cDNA synthesis and predenaturation (1 cycle at 55 °C for 30 min followed by 94 °C for 2 min); PCR amplification (30 cycles of denaturing at 94 °C for 15 s, annealing at 55 °C for 30 s and extension at 68 °C for 60 s) and a final extension at 68 °C for 5 min. The PCR products were electrophoresed on 2% agarose gels and visualized with Nancy-520 (Sigma) under ultraviolet light. All primers were synthesized by Eurofins. Sequences of these primers are: MLK2 forward (5′-GAACGTGAGATGGACATCGTGGAA-3′), MLK2 reverse (5′-AGGCCTGGACTGTGATCTTATGCT-3′), GAPDH forward (5′-CCATGGAGAAGGCTGGGG-3′) and GAPDH reverse (5′-GTCCACCACCCTGTTGCTGTA-3′).

### Statistical and structural analysis

For xenograft studies, MTT and annexin V/7-AAD tests, statistical comparison of values was performed using the Student’s *t*-test. The homology model of the MLK1 D176N kinase domain was created using SwissModel^36^ using the MLK1 kinase domain as a template (PDB accession code: 3DTC). Analysis of polar contacts was performed within PyMol v1.3.

## Additional information

**How to cite this article**: Marusiak, A. A. *et al.* Mixed lineage kinases activate MEK independently of RAF to mediate resistance to RAF inhibitors. *Nat. Commun.* 5:3901 doi: 10.1038/ncomms4901 (2014).

## Supplementary Material

Supplementary Information Supplementary Figures 1-6 and Supplementary Table 1
