# Supplementary material for: Mixed lineage kinases activate MEK independently of RAF to mediate resistance to RAF inhibitors
Source: Nat Commun. 2014 May 22;5:3901. doi: 10.1038/ncomms4901 (PMC4046110; doi:10.1038/ncomms4901)
Supplement: Supplementary Information — Supplementary Figures 1-6 and Supplementary Table 1 [file ncomms4901-s1.pdf]

Supplementary Information

Supplementary Figures

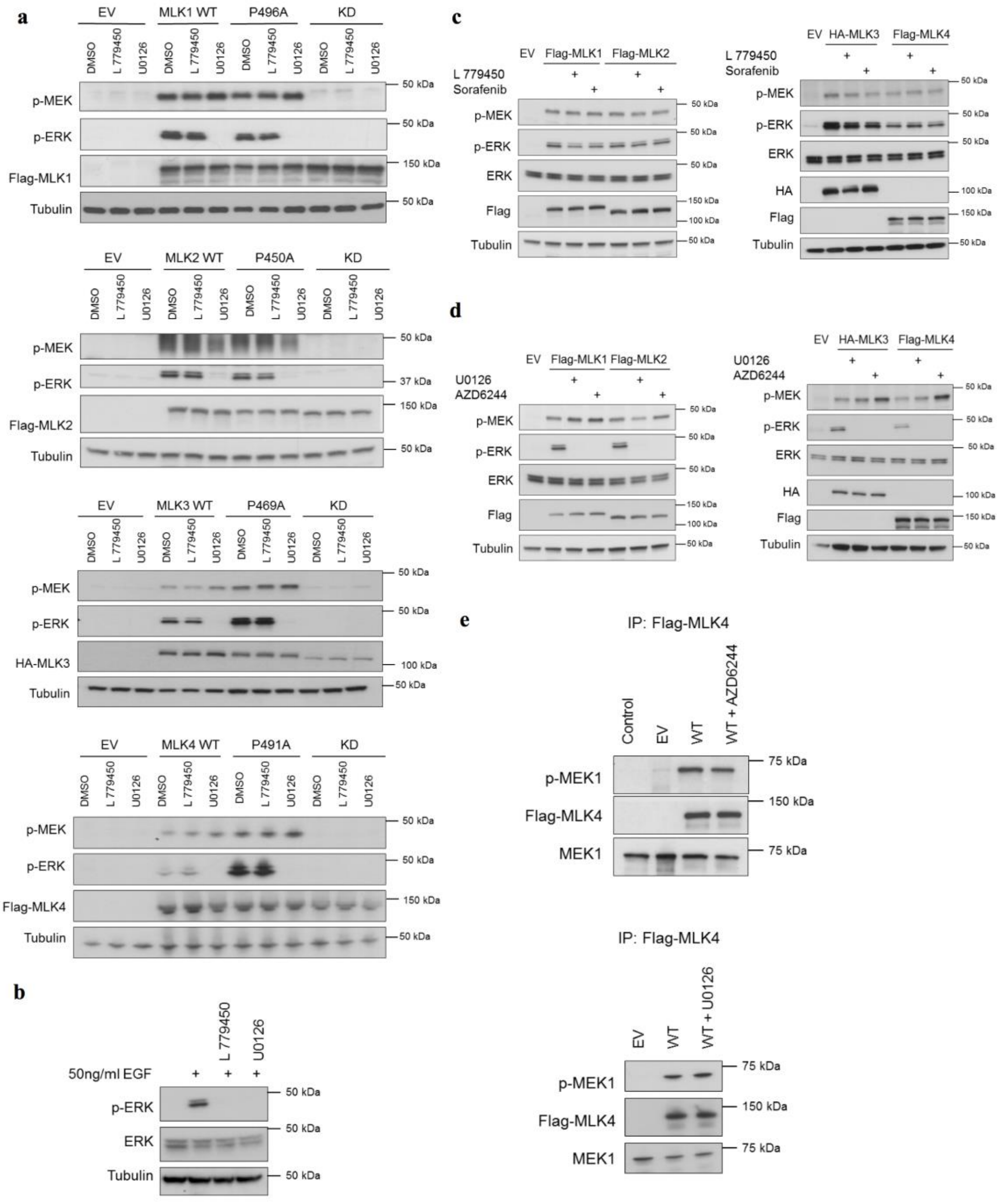

Supplementary Figure 1. MLK1-4 phosphorylate MEK in the presence of RAF inhibitors. (**a**) H157 cells were transiently transfected with Flag- or HA-tagged MLK1-4 constructs or EV (empty vector) control. Cells were treated with 5 μM L779450, 5 μM U0126 or DMSO for 1 hour and lysed. Whole cell lysates were analyzed by western blot. (**b**) L779450 and U0126 inhibit ERK

phosphorylation. HEK293T cells were treated with 5 μM L779450 or 5 μM U0126 for 1 hour before stimulation with 50 ng/ml EGF for 10 minutes. Whole cell lysates were analyzed by western blot. (**c**) HEK293T cells were transiently transfected with Flag- or HA-tagged MLK1-4 WT or EV. After 48 hours cells were treated with 5 μM L779450 or 10 μM sorafenib for 1 hour and lysed. Cell lysates were analyzed by western blot. (**d**) MLK1-4 ERK pathway activation is abolished in the presence of MEK inhibitors. HEK293T cells were transiently transfected with Flag- or HA-tagged MLK1-4 WT or EV. After 48 hours cells were treated with 5 μM U0126 or 2 μM AZD6244 for 1 hour and lysed. Whole cell lysates were analyzed by western blot. (**e**) Flag-MLK4β WT or EV control were transiently transfected in HEK293T cells. Cells were treated with 5 μM U0126, 2 μM AZD6244 or DMSO for 1 hour before lysis. MLK4 was immunoprecipitated and subjected to kinase assay using kinase inactive MEK1. Control sample does not include any cell lysate. All results are representative of three independent experiments.

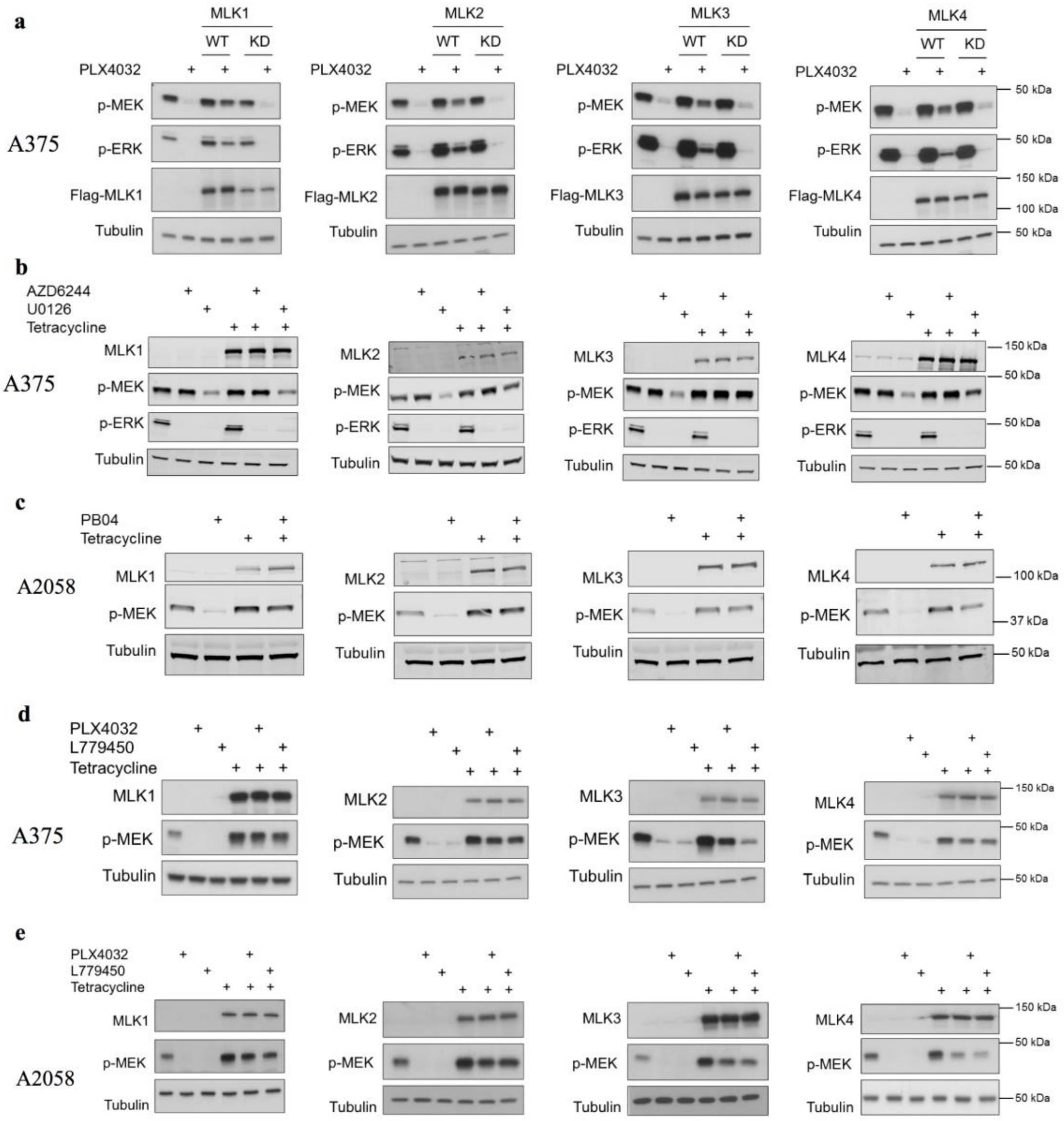

Supplementary Figure 2. MLK1-4 reactivate the MEK/ERK pathway in V600E-positive melanoma cells independently of the RAF kinases. (**a**) Kinase-dependent phosphorylation of the MEK/ERK pathway in A375 cells expressing MLKs in the presence of vemurafenib. A375 cells were transiently transfected with Flag-MLK1-4 WT (wild type), KD (kinase dead) or EV (empty vector). 48 hours later cells were treated with 1 μM PLX4032 (vemurafenib) or DMSO for 1 hour followed by lysis. Cell lysates were analyzed by western blot. (**b**) MLKs expression does not reactivate the MEK/ERK pathway in the presence of MEK inhibitors. Expression of MLKs was induced by tetracycline. The next day A375 cells were treated with MEK inhibitors (5 μM U0126 and 2 μM AZD6244) or DMSO for 1 hour. Cell lysates were analyzed by western blot. (**c**) Expression of

MLK1-4 in A2058 cells was induced by addition of tetracycline. After 24 hours cells were treated with 1 μM PB04 or DMSO for 1 hour, lysed and analyzed by western blot. (**d, e**) Overexpression of MLKs in melanoma cells treated with PLX4032 (vemurafenib) or pan-RAF kinase inhibitor leads to reactivation of the MEK/ERK pathway. Expression of MLKs in (**d**) A375 or (**e**) A2058 cells was induced by addition of tetracycline. 24 hours later cells were treated with 1 μM PLX4032, 5 μM L779450 or DMSO for 1 hour, lysed and analyzed by western blot. All results are representative of three independent experiments.

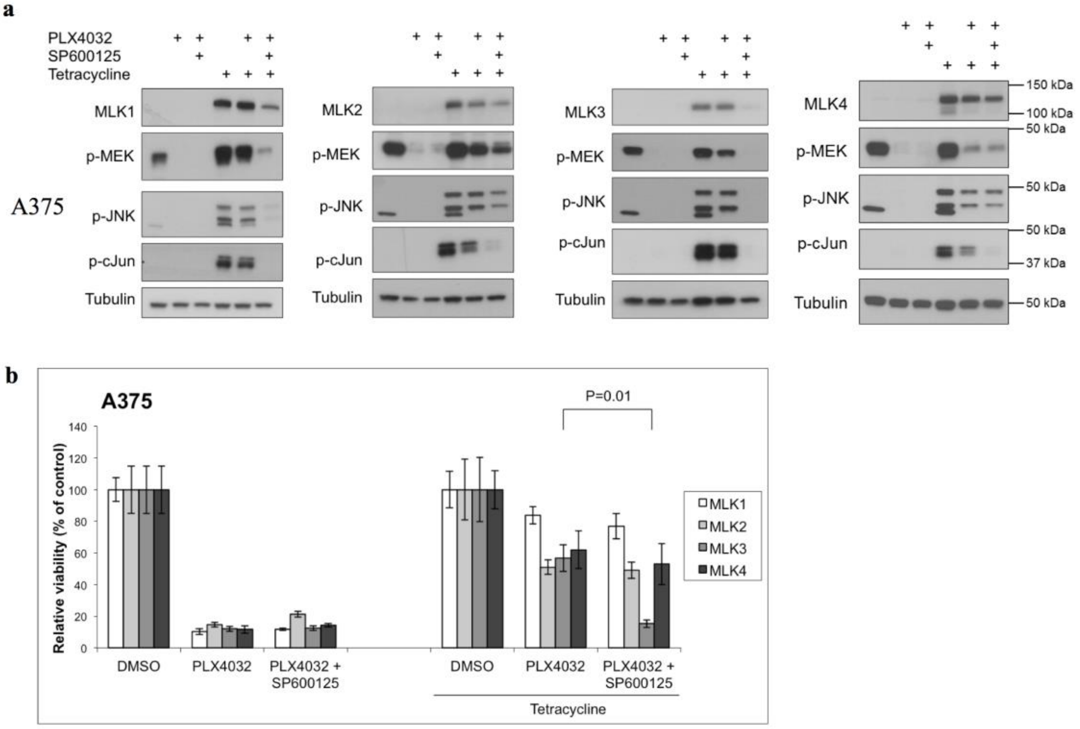

Supplementary Figure 3. JNK pathway inhibition does not influence the survival of cells expressing MLKs. (**a**) Expression of MLKs in A375 cells was induced by addition of tetracycline followed by treatment with 1 μM PLX4032 alone or together with 20 μM SP600125 for 18 hours, lysed and analyzed by western blot. All results are representative of three independent experiments. (**b**) Expression of MLKs in A375 cells was induced by tetracycline followed by treatment with 1 μM PLX4032 alone or together with 20 μM SP600125 for 4 days. Cell viability was determined by MTT assay. Error bars indicate ±SEM from three independent experiments performed in triplicate (n = 9). P values calculated by Student's t-test.

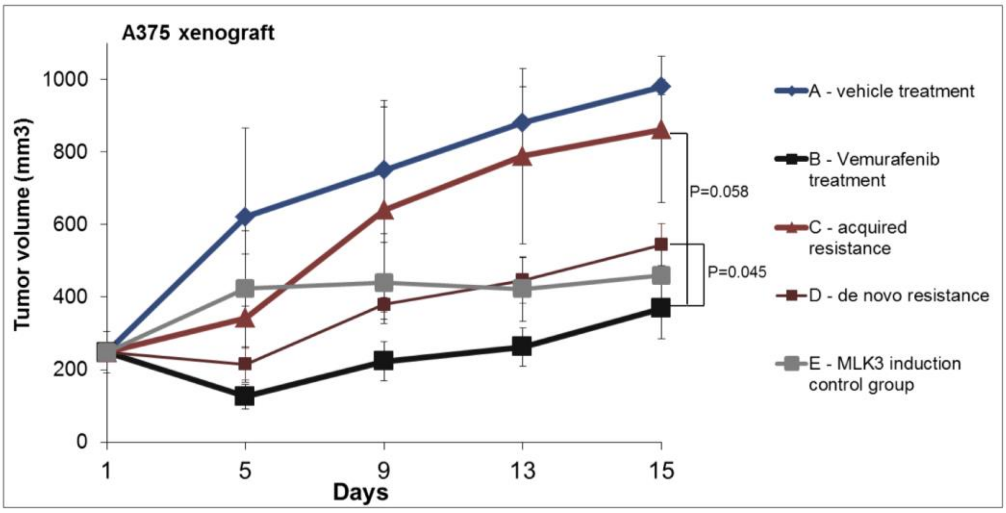

Supplementary Figure 4. Induced expression of MLK3 promotes tumor survival in a xenograft mouse model. A375 cells were engrafted in SCID mice. After tumor establishment (day 1), mice were treated with vehicle (groups A and E) or vemurafenib (25 mg per kg) (group B, C and D). MLK3 expression by doxycycline induction started on the same day for group D and E or 5 days later for group C (day 5). Mean tumor volumes ±SEM are shown (n = 3 mice per group).

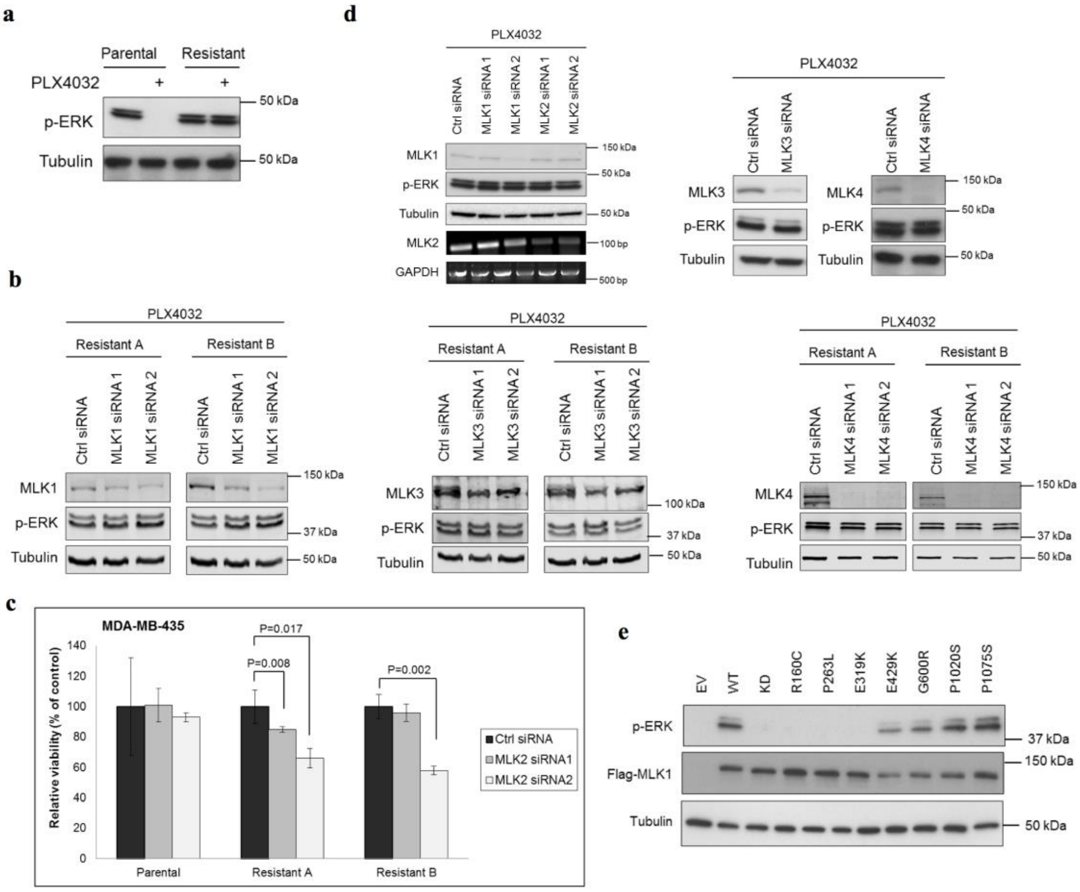

Supplementary Figure 5. MLKs promote resistance to vemurafenib. (**a**) Vemurafenib-resistant A375 cells. A375 cells, parental and drug-resistant, were seeded in 12 well plates. Drug-resistant cells were seeded in the presence of vemurafenib. Next day cells were treated with vemurafenib for 1 hour. Cell lysates were analyzed by western blot. (**b**) MLK1, 3 or 4 knockdown does not reduce ERK signaling in vemurafenib-resistant MDA-MB-435 cells. MDA-MB-435 vemurafenib-resistant clones A and B were transfected with siRNA against MLK1, MLK3 and MLK4 or control siRNA and lysed after 48 hours. Resistant A and B were grown in the presence of vemurafenib. Activation of the MEK/ERK pathway was analyzed by western blot. (**c**) MLK2 depletion causes reduction in cell viability in resistant MDA-MB-435 cells. MDA-MB-435 parental and resistant clones A and B were transfected with siRNA against MLK2 or control. Resistant A and B were grown in the presence of vemurafenib. Cell viability was determined by MTT assay after 2 days. Error bars indicate ±SEM from three independent experiments performed in triplicate (n = 9). P values calculated by Student's t-

test. (**d**) MLK1-4 knockdown does not affect vemurafenib-resistant A375 cells. Drug-resistant A375s were transfected with siRNA against MLK1-4 or control siRNA and lysed after 48 hours. Resistant cells were grown in the presence of vemurafenib. Activation of the MEK/ERK pathway and expression levels of MLK1, MLK3 and MLK4 were analyzed by western blot and the level of MLK2 was verified by RT-PCR. (**e**) MLK1 loss-of-function and neutral mutations. H157 cells were transiently transfected with EV (empty vector), Flag-MLK1 WT (wild type), KD (kinase dead) or MLK1 mutants. After 48 hours cells were lysed and cell lysates were analyzed by western blot. All results are representative of three independent experiments.

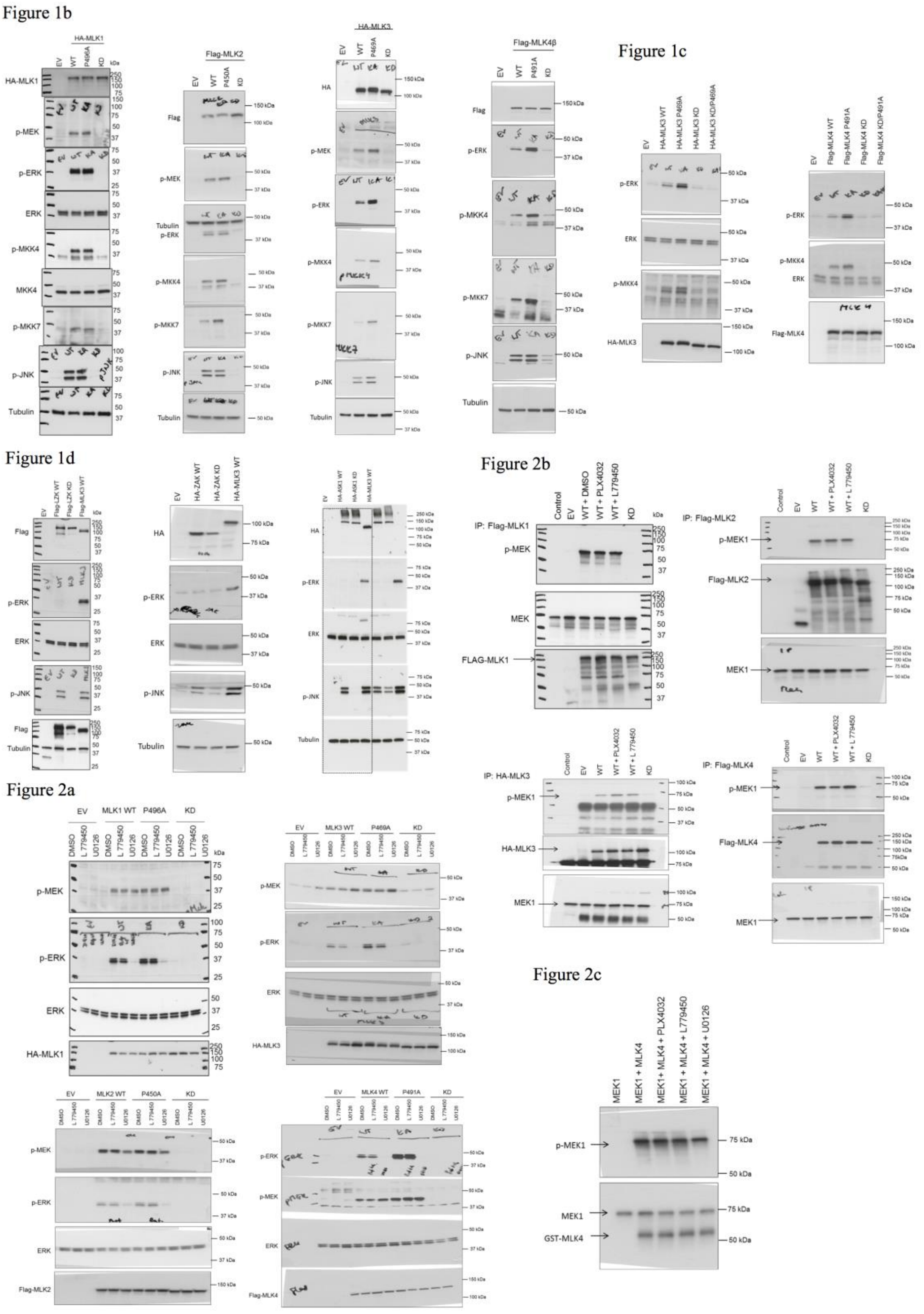

Supplementary Figure 6. Uncropped scans of the most important immunoblots.

Figure 3a

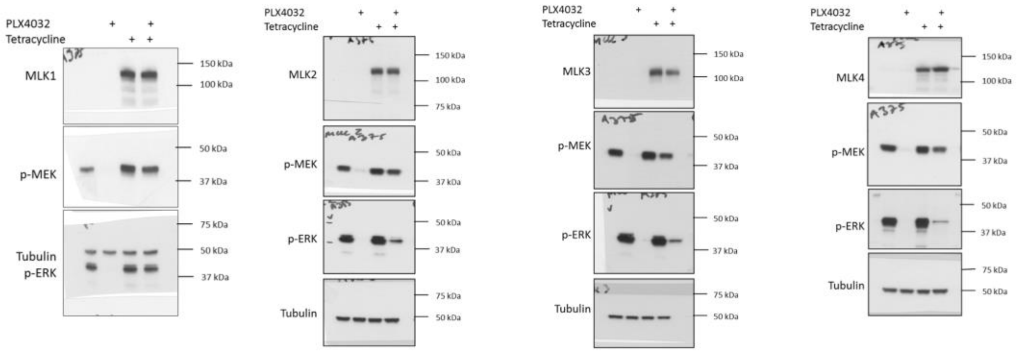

Figure 3b

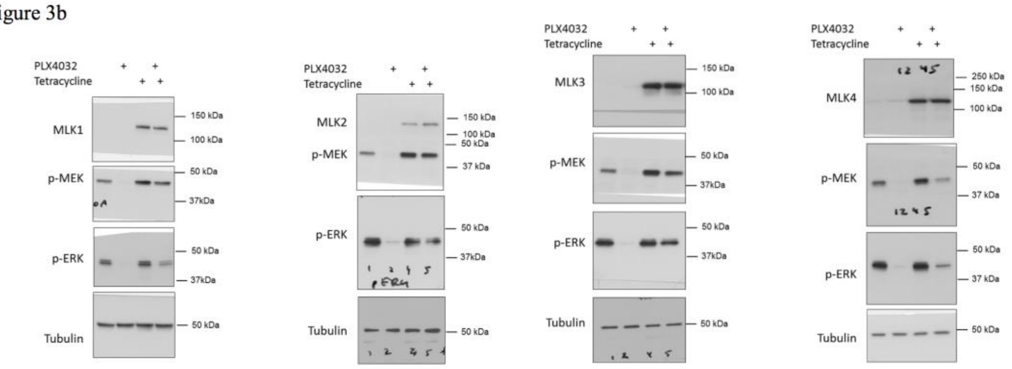

Figure 3c

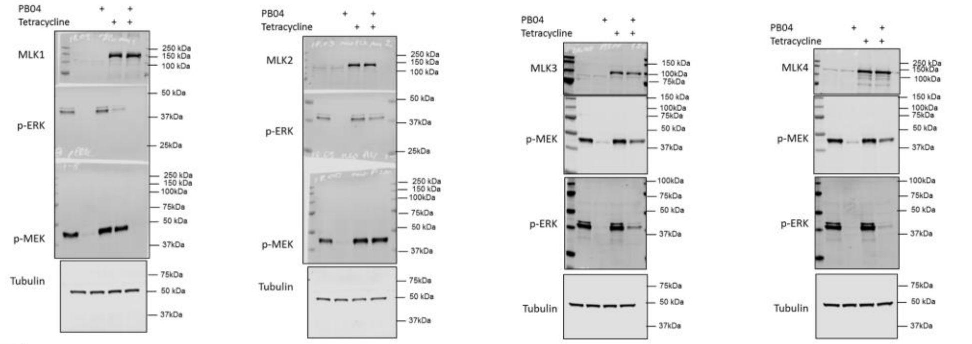

Figure 3d

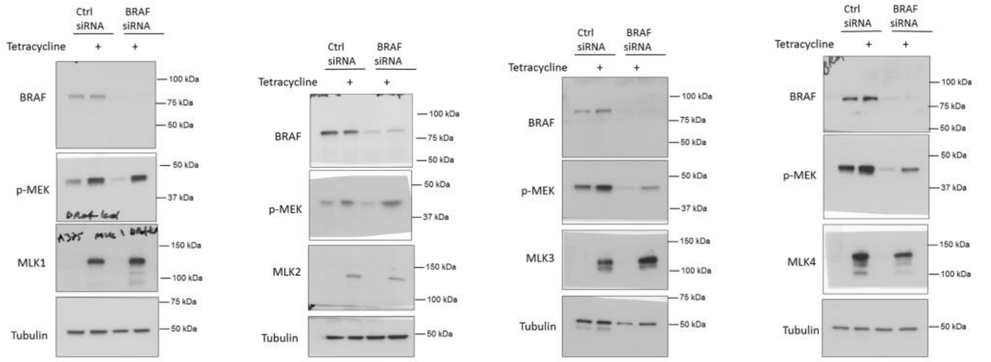

Supplementary Figure 6 continued

Figure 4c

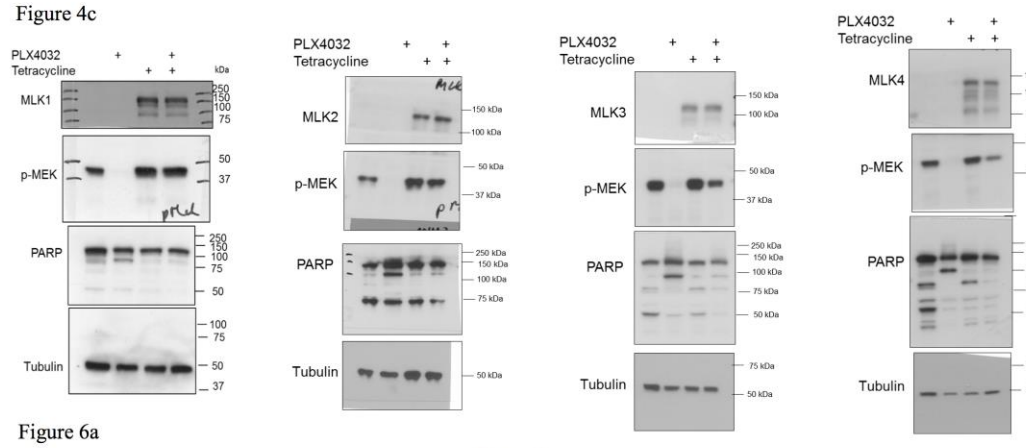

Figure 6a

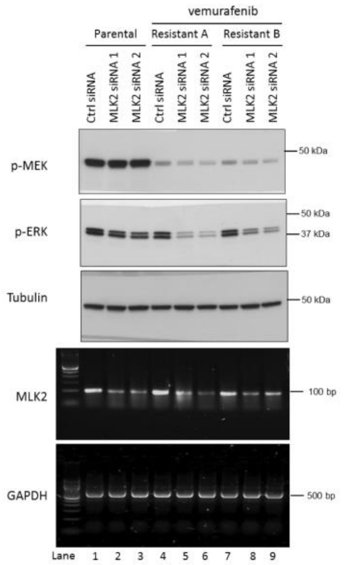

Figure 6b

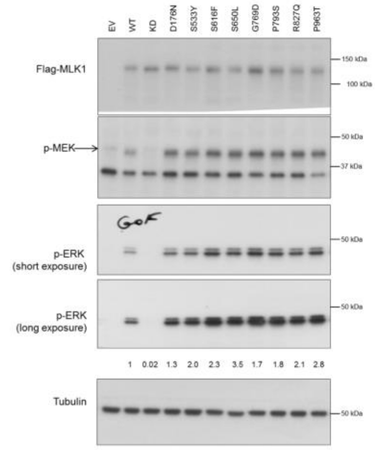

Supplementary Figure 6 continued

Supplementary Table 1. The recurrence of MLK1-4 upregulations in a set of 29 disease progression (DP) biopsies from 21 patients (P) together with the status of MAPK pathway activation and resistance characteristics

Resistant tumor IDs	COT	MLK1	MLK2	MLK3	MLK4	MAPK activity#	Resistance mechanism#
P1-DP1-Dab						+	*MEK1*^{E203K}
P2-DP1-Dab						+	*NRAS*^{Q61K}
P3-DP1-Dab						+	Unknown
P3-DP2-Dab						+	Unknown
P4-DP1-Dab						+	*BRAF* exon 2-10Δ
P5-DP1-Dab						+	Unknown
P7-DP1-Dab						+	*BRAF* exon 4-8Δ
P8-DP1-Dab						+	Unknown
P8-DP2-Dab						+	Unknown
P9-DP1-Dab						+	*BRAF* exon 2-10Δ
P10-DP1-Dab						-	IGF-IR
P10-DP2-Dab						+	*BRAF* exon 4-8Δ
P11-DP1-Dab						-	Unknown
P11-DP2-Dab						+	*AKT1*^{Q79K}
P12-DP1-Dab						+	Unknown
P13-DP1-Dab						-	Unknown
P14-DP1-Dab						+	*BRAF* amplification
P17-DP1-Dab						+	Unknown
P18-DP1-Dab						-	Unknown
P18-DP2-Dab						+	*MEK1*^{K57E}
P21-DP1-Dab						-	Unknown
P23-DP1-Vem						+	*BRAF* exon 2-8Δ
P24-DP1-Vem						+	*NRAS*^{G13Rf}
P25-DP1-Vem						-	Unknown
P28-DP1-Vem						+	*BRAF* exon 2-8Δ
P28-DP2-Vem						+	*BRAF* exon 4-8Δ
P28-DP3-Vem						+	*BRAF* exon 2-8Δ
P28-DP4-Vem						+	*BRAF* exon 4-8Δ
P30-DP1-Vem						+	*MEK2*^{F57C}

Rizos et al, Clin Cancer Res, 2014
Dab – dabrafenib, Vem – vemurafenib
Tiles with color shading signify statistically significant upregulation of the gene expression (fold-change ≥ 2 (black) or 1.5 ≤ fold-change ≤ 2 (grey)) in the DP samples as compared to their respective baselines
